# Quantification of Circulating Cell-Free DNA in Idiopathic Parkinson’s Disease Patients

**DOI:** 10.3390/ijms25052818

**Published:** 2024-02-29

**Authors:** Małgorzata Wojtkowska, Natalia Karczewska, Klaudia Pacewicz, Andrzej Pacak, Piotr Kopeć, Jolanta Florczak-Wyspiańska, Karolina Popławska-Domaszewicz, Tomasz Małkiewicz, Bartosz Sokół

**Affiliations:** 1 Department of Bioenergetics, Faculty of Biology, Adam Mickiewicz University, 61-614 Poznan, Poland; klaudia.pacewicz1@gmail.com; 2 Centre for Chemical Biology, Institute of Bioorganic Chemistry, Polish Academy of Sciences, 61-704 Poznan, Poland; nkarczewska@ibch.poznan.pl; 3 Department of Gene Expression, Faculty of Biology Poznan, Adam Mickiewicz University, 61-614 Poznan, Poland; pacak@amu.edu.pl; 4 Department of Computational Biology, Faculty of Biology, Adam Mickiewicz University, 61-614 Poznan, Poland; piotr.kopec@amu.edu.pl; 5 Department of Neurology, Clinical Hospital of Poznan University of Medical Sciences, 60-355 Poznan, Poland; jolaflorczak@op.pl (J.F.-W.); kpoplawska@ump.edu.pl (K.P.-D.); 6 Department of Teaching Anaesthesiology and Intensive Therapy, Poznan University of Medical Sciences, 60-355 Poznan, Poland; tmalkiewicz@gmail.com; 7 Department of Neurosurgery, Poznan University of Medical Sciences, 60-355 Poznan, Poland; bartosz.sokol@gmail.com; 8 Hospital of Joseph Strus in Poznan, 61-285 Poznan, Poland

**Keywords:** Parkinson’s disease, mitochondria, circulating cell free DNA (ccf-DNA), mitochondrial (mtDNA), nuclear (ccfDNA), droplet digital PCR (ddPCR)

## Abstract

Parkinson’s disease (PD) is one of the most common neurodegenerative disorders globally and leads to an excessive loss of dopaminergic neurons in the substantia nigra of the brain. Circulating cell-free DNA (ccf-DNA) are double-stranded DNA fragments of different sizes and origins that are released into the serum and cerebrospinal fluid (CSF) due to cell death (i.e., necrosis and apoptosis) or are actively released by viable cells via exocytosis and NETosis. Using droplet digital polymerase chain reaction (ddPCR), we comprehensively analyzed and distinguished circulating cell-free mitochondrial DNA (ccf mtDNA) and circulating cell-free nuclear DNA (ccfDNA) in the serum and CSF of PD and control patients. The quantitative analysis of serum ccf-DNA in PD patients demonstrated a significant increase in ccf mtDNA and ccfDNA compared to that in healthy control patients and a significantly higher copy of ccf mtDNA when compared to ccfDNA. Next, the serum ccf mtDNA levels significantly increased in male PD patients compared to those in healthy male controls. Furthermore, CSF ccf mtDNA in PD patients increased significantly compared to ccfDNA, and ccf mtDNA decreased in PD patients more than it did in healthy controls. These decreases were not statistically significant but were in agreement with previous data. Interestingly, ccf mtDNA increased in healthy control patients in both serum and CSF as compared to ccfDNA. The small sample size of serum and CSF were the main limitations of this study. To the best of our knowledge, this is the first comprehensive study on serum and CSF of PD patients using ddPCR to indicate the distribution of the copy number of ccf mtDNA as well as ccfDNA. If validated, we suggest that ccf mtDNA has greater potential than ccfDNA to lead the development of novel treatments for PD patients.

## 1. Introduction

Parkinson’s disease (PD) is the second most common neurodegenerative disease globally. It leads to the excessive loss of dopaminergic neurons in the substantia nigra of the brain. Accurate diagnosis of the disease remains challenging, and methods of characterizing the earliest stages of the disease is a focus of ongoing research [1,2]. Like in many neurodegenerative diseases, PD symptoms occur well after pathology begins due to the compensatory potential of the brain. Furthermore, due to the massive death of neuronal cells, it is difficult to treat advanced-stage PD patients. Therefore, a simple and non-invasive method of early diagnosis could increase the efficiency of the limited treatment options available [1,2,3,4].

Circulating cell-free DNA (ccf-DNA) are short, double-stranded DNA fragments present in various body fluids, such as the blood, urine, serum, and cerebrospinal fluid (CSF) [5,6,7,8]. Depending on the type of DNA released, ccf-DNA has two main sources: nuclear (ccfDNA) and mitochondrial (ccf mtDNA). It is believed that ccf-DNA is released due to cell death (i.e., necrosis or apoptosis) and through active release by viable cells via exocytosis and NETosis [9,10,11,12]. For other possible mechanisms of ccf mtDNA’s stress-induced release and its physiological considerations, see an elegant review [13]. For the last decade, ccf-DNA has become a subject of interest for the non-invasive analysis of tumor-derived genetic material. Both ccfDNA and mtDNA have been the focus of qualitative and quantitative investigations. Alterations in these two types of ccf-DNA have also been implicated in various types of cancer [14,15,16,17]. 

Limited reports have primarily focused on the quantification of ccf-DNA levels in CSF of PD patients. Most studies involving ccf DNA have focused on ccf mtDNA [18,19], and post-mortem studies [20]. In these studies, ccf mtDNA has been found in decreased levels among PD patients compared to healthy controls. Lowes (2020a) found that a positive correlation existed between CSF ccf mtDNA and various comorbidities such as depression and insomnia, however this was only significant if measured in the absence of treatment [19,21]. Regarding serum, the study by Borsche et al. (2020) investigated the sporadic form of PD and found that patients with biallelic PINK1 and Parkin mutations had elevated levels of ccf mtDNA and IL-6, suggesting increased ccf mtDNA release and neuroinflammation in these PD patients [22]. There are currently no studies presenting quantification of the serum and CSF ccf mtDNA or ccfDNA of idiopathic PD patients. Such data could be useful for diagnostic purposes and could provide better understanding of the association between serum and CSF ccf-DNA and neurodegeneration. 

In this study, we performed quantitative and qualitative studies on ccf-DNA isolated from the serum and CSF of idiopathic PD and healthy control patients using droplet digital PCR (ddPCR). This method allowed for the precise specification of the copy number of ccf mtDNA and ccfDNA. We also correlated the level of ccf-DNA with gender in the studied groups.

## 2. Results

Our aim was to characterize ccf-DNA in the serum and CSF of PD patients to compare them against healthy control patients. To distinguish differentially derived DNA, we chose target DNA fragments of the mitochondrial *COX3*, a gene encoding mitochondrial cytochrome c oxidase III, and nuclear *KRAS*, an oncogene encoding a protein belonging to the GTPase superfamily. These have previously been used in analogue studies involving different groups of patients [23,24]. Thus, we analyzed ccf-DNA isolated from the serum of 30 PD and 15 healthy control patients and the CSF from 13 PD and five healthy control patients. The PD patient cohort was selected by neurologists from the Department of Neurology University of Medical Sciences Poznan, Poland, from patients diagnosed with PD (Table 1).

ddPCR was used here to enable analysis of small amounts of material. Unlike quantitative PCR (qPCR), ddPCR does not require reference gene copy analysis [25,26]. In ddPCR, amplification occurs in small volume (1 nL) droplets. The total number of droplets is then calculated as the number of positive droplets (i.e., those that fluoresce) less the number of negative droplets (i.e., those with no signal). These are counted in a flow cytometry-like fashion to produce a ratio that is then subjected to Poisson distribution, resulting in the absolute quantification of starting template molecules [27,28]. The copy numbers of ccf mtDNA and ccfDNA were calculated in this manner and underwent statistical analysis using the Mann–Whitney U test.

Methods of quantifying mtDNA copy numbers with ddPCR using purified genomic DNA have been developed previously [23,27,29,30,31]. Here, ccfDNA was purified immediately after blood donation by isolation from the frozen serum and CSF of 0.2 mL samples according to the Qiagen procedure. 

### 2.1. Quantification of Serum ccf-DNA in PD Patients 

#### Serum ccf mtDNA and ccfDNA in PD Patients versus Control Patients

**Figure 1:**
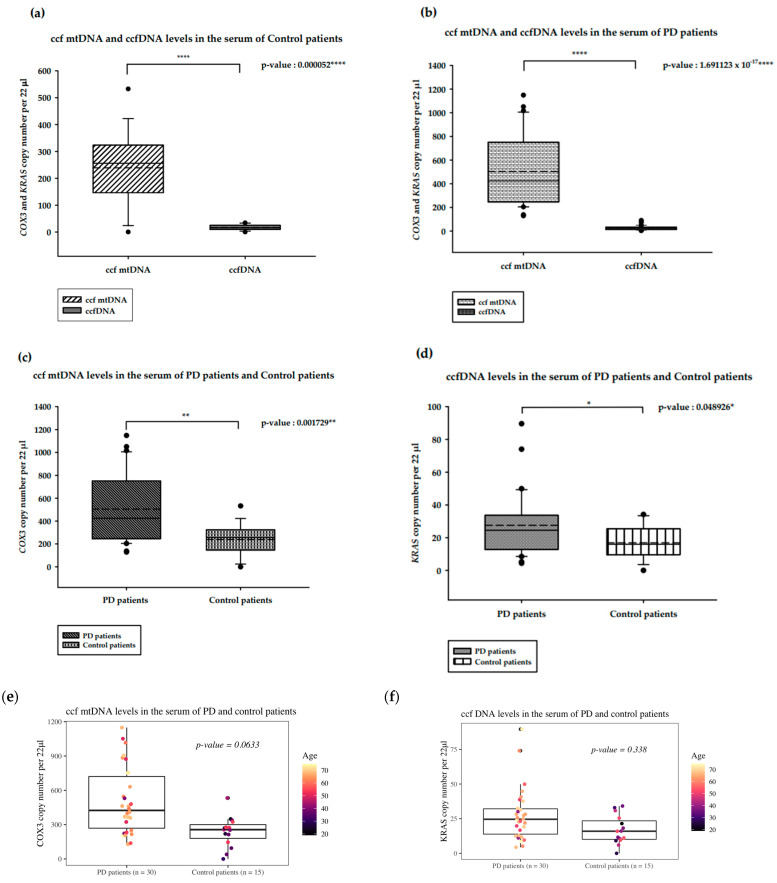
Results of the ddPCR analysis on serum ccf DNA from PD and healthy controls. (**a**). ccf mtDNA and ccfDNA copy numbers in the serum of healthy controls; (**b**). ccf mtDNA and ccfDNA copy numbers in the serum of PD patients; (**c**). ccf mtDNA copy numbers in the serum of PD and healthy controls; (**d**). ccfDNA copy numbers in the serum of PD and healthy controls; Boxplots represent medians (solid lines) and means (dashed lines) with min and max values. Mann–Whitney U tests were performed for comparison. A probability of ≤0.05 was considered statistically significant; * *p* ≤ 0.05, ** *p* ≤ 0.01, **** *p* ≤ 0.0001 (**e**). ccf mtDNA copy numbers in the serum of PD and healthy controls compared with ANOVA with age as a covariate; (**f**). ccfDNA copy numbers in the serum of PD and healthy controls compared with ANOVA with age as a covariate.

We analyzed and compared the ccf DNA isolated from PD and healthy control patients, as shown in Figure 1. In the serum from the control patients, ccf mtDNA levels were significantly higher than the ccfDNA levels (*p*-value = 0.000052 ****; Figure 1a). In PD patients, ccf mtDNA levels were higher than ccfDNA levels (*p*-value: 1.691123 × 10^−17^; Figure 1b). The ccf mtDNA levels of PD patients were significantly increased compared to those of the control patients (*p*-value = 0.001729 **; Figure 1c). Interestingly, ccfDNA levels among PD patients were also significantly higher than those in the control patients (*p*-value = 0.0048926 *; Figure 1d). All presented analyses were statistically significant. 

Because the age of the PD and control healthy patients differs (median 37 and 66, respectively) we have performed ANOVA analysis with age as a covariant to assess the relationship of the Parkinson’s disease presence and ccf mtDNA. In this case, multiple linear regression was performed. The overall association was statistically significant (F = 5.542, *p* = 0.007309), with the model explaining 17.11% of the variance. However, the observed increase of the ccf mtDNA copy number among PD patients was not statistically significant at α = 0.05 (t = 1.908, *p* = 0.0633; Figure 1e). The obtained results revealed that age is not associated with ccf mtDNA levels in PD versus control healthy patients (t = 0.362, *p* = 0.719; Figure 1f). Analogous analysis was conducted for ccfDNA levels, although there was no significant overall association (F = 2.173, *p* = 0.1264) (Supplementary File S2 Tables S1 and S2). 

### 2.2. Quantification of CSF ccf-DNA in PD Patients 

#### CSF ccf mtDNA and ccfDNA in PD Patients versus Healthy Controls

**Figure 2:**
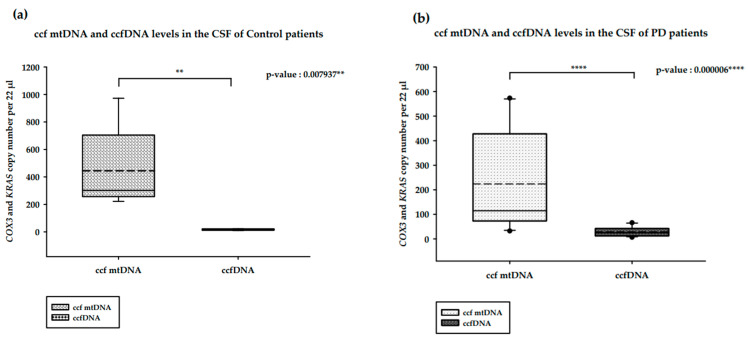

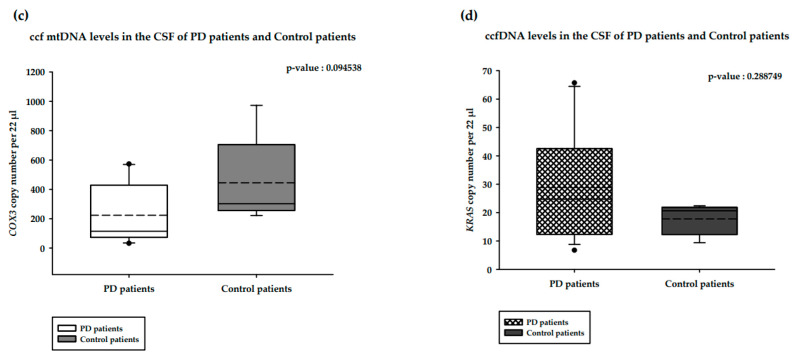
Results of the ddPCR analysis on the CSF ccfDNA of PD patients and healthy controls. (**a**). ccf mtDNA and ccfDNA copy numbers in the CSF of healthy controls; (**b**). ccf mtDNA and ccfDNA copy numbers in the CSF of PD patients; (**c**). ccf mtDNA copy numbers in the CSF of PD patients and healthy controls; (**d**). ccfDNA copy number in the CSF of PD patients and healthy controls. Boxplots represent medians (solid lines) and means (dashed lines) with min and max values. Mann–Whitney U tests were performed for comparison. A probability of ≤0.05 was considered statistically significant; ** *p* ≤ 0.01, **** *p* ≤ 0.0001.

We analyzed and compared the ccfDNA isolated from the CSF of PD and healthy controls (Figure 2). In the CSF of healthy controls, ccf mtDNA levels were significantly higher than ccfDNA levels (*p*-value = 0.007937 **; Figure 2a). The same pattern was observed in PD patients, with the difference also being significant (*p*-value = 0.000006 ****; Figure 2b). In contrast to the results obtained for the serum levels, CSF ccf mtDNA levels among PD patients were lower than in the control patients (*p*-value = 0.094538; Figure 2c). ccfDNA levels were higher in PD patients compared to control patients; But, this difference was not statistically significant (*p*-value = 0.288749; Figure 2d).

### 2.3. Distribution of the Copy Number of Serum ccf-DNA and Gender in PD Patients

#### 2.3.1. Distribution of the Copy Number of Serum ccf mtDNA and Gender in PD versus Healthy Controls

**Figure 3:**
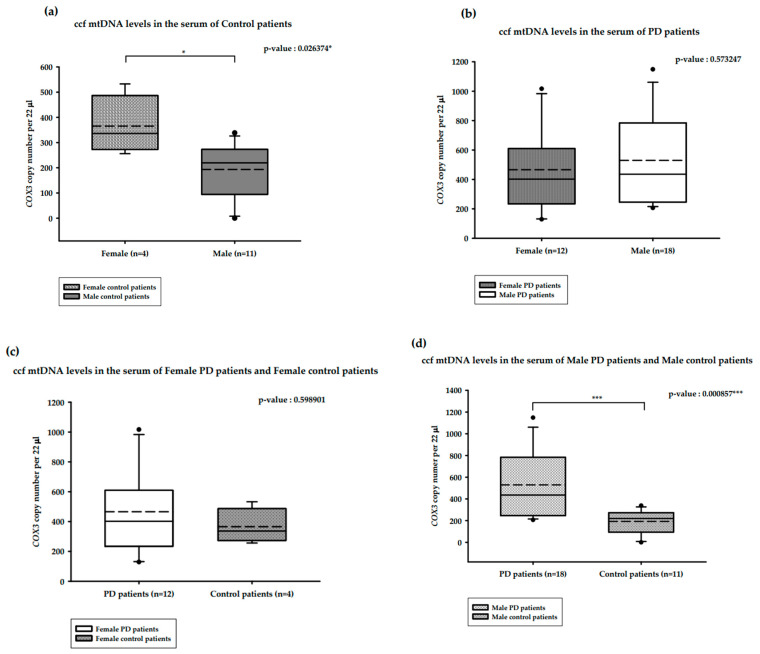
Distribution of copy numbers of serum ccf mtDNA and gender in PD patients versus healthy controls. (**a**). ccf mtDNA copy numbers in the serum of female and male controls; (**b**). ccf mtDNA copy numbers in female and male PD patients; (**c**). ccf mtDNA copy numbers in female PD patients and female controls; (**d**). ccf mtDNA copy numbers in male PD patients and male controls. Boxplots represent medians (solid lines) and means (dashed lines) with min and max values. Mann–Whitney U tests were performed for comparison. A probability of ≤0.05 was considered statistically significant; * *p* ≤ 0.05, *** *p* ≤ 0.001.

The correlation between serum ccf mtDNA and gender is presented in Figure 3. The median serum ccf mtDNA copy numbers among healthy males (*n =* 11) and females (*n =* 4) were 219.73 and 336.43 copies/22 µL, respectively. This difference was statistically significant, with increased copy numbers observed among the healthy females (Mann–Whitney U test, *p*-value = 0.026374 *; Figure 3a). The median serum ccf mtDNA copy numbers among female PD patients (*n =* 12) and male PD patients (*n =* 18) were 402 and 435.47 copies/22 µL, respectively, although this difference was not statistically significant (Mann–Whitney U test, *p*-value = 0.573247; Figure 3b). The median serum ccf mtDNA copy numbers among female PD patients (*n =* 12) and healthy females (*n =* 4) were 402 and 336.43 copies/22 µL, respectively, and this difference was also not statistically significant (Mann–Whitney U test, *p*-value = 0.598901; Figure 3c). The median serum ccf mtDNA copy numbers among male PD patients (*n =* 18) and healthy males (*n =* 11) were 435.47 and 219.73 copies/22 µL, respectively. This difference was statistically significant, with double the copy numbers seen in male PD patients (Mann–Whitney U test, *p*-value = 0.000857 ***; Figure 3d). 

#### 2.3.2. Distribution of the Copy Number of Serum ccfDNA and Gender in PD versus Healthy Controls

**Figure 4:**
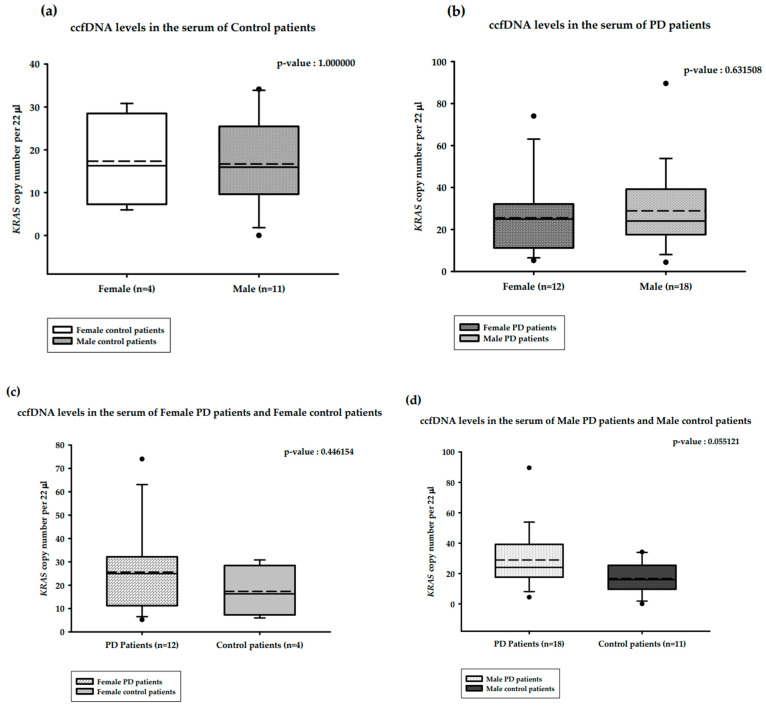
Distribution of copy numbers of serum ccfDNA and gender of PD patients versus healthy controls. (**a**). ccfDNA copy numbers in the serum of female and male controls; (**b**). ccfDNA copy numbers in female and male PD patients; (**c**). ccfDNA copy numbers in the serum of female PD patients and female controls; (**d**). ccfDNA copy number in male PD Patients and male controls. Boxplots represent medians (solid line) and means (dashed line) with min to max values. Mann–Whitney U tests were performed for comparison. A probability of ≤0.05 was considered statistically significant.

The correlations between serum ccfDNA and gender are presented in Figure 4. The median serum ccfDNA copy numbers in the healthy male (*n =* 11) and healthy female (*n =* 4) groups were 15.94 and 16.28 copies/22 µL, respectively. This difference was not statistically significant (Mann–Whitney U test, *p*-value = 1.000000; Figure 4a). The median serum ccfDNA copy numbers among female PD patients (*n =* 12) and male PD patients (*n =* 18) were 24.95 and 24.02 copies/22 µL, respectively, and this was also not a statistically significant difference (Mann–Whitney U test, *p*-value = 0.631508; Figure 4b). The median serum ccfDNA copy numbers in female PD patients (*n =* 12) and healthy females (*n =* 4) were 24.95 and 16.28 copies/22 µL, respectively; this difference was not statistically significant (Mann–Whitney U test, *p*-value = 0.446154; Figure 4c). The median serum ccfDNA copy numbers in male PD patients (*n =* 18) and healthy males (*n =* 11) were 24.02 and 15.94 copies/22 µL, respectively; this difference was statistically significant (Mann–Whitney U test, *p*-value = 0.055121; Figure 4d). 

## 3. Discussion

To the best of our knowledge, this is the first comprehensive study of the serum and CSF of idiopathic PD patients performed using the precise technique of ddPCR to enable presentation of the distribution of the copy number of both ccf mtDNA and ccfDNA. Our data obtained for the serum reveal a significant increase in the copy number of ccf mtDNA versus healthy control patients. For both the serum and the CSF, a higher copy number of the ccf mtDNA compared to ccfDNA was observed (Table 2). In previous studies, it has been shown that mitochondria can control inflammation through the production of reactive oxygen species (ROS) and the release of mitochondrial components, including mitochondrial DNA (mtDNA), into the extracellular matrix, where they act as danger signals [32]. We found that ccf mtDNA dominated in the serum of PD patients; however, this does not reflect our observation regarding CSF, where ccf mtDNA is reduced. Other studies have shown a reduced level of mtDNA in leucocytes of PD patients compared to that in healthy controls [33]. We suggest this could reflect reduced numbers of mitochondria in the blood of PD patients. Thus, the increased copy number of mitochondrially derived ccf-DNA presented by us could be explained as a consequence of the unknown mechanism of selective mtDNA degradation that leads to stress-induced ccf mtDNA release. It could also suggest evidence of the potential pro-inflammatory effects of blood ccf mtDNA that definitely need further study.

Interestingly, unlike nuclear DNA, mtDNA contains unmethylated CpG sequences (a pattern common to bacterial DNA), which act as damage-associated molecular patterns (DAMP) [34,35]. It is believed that this ccf mtDNA allows non-self-recognition, further contributing to immune system activation [34] and stimulating an innate immune response through a variety of receptors expressed in neurons [36,37,38] and an inflammatory response described for (PRKN-PINK) PD patients [39]. According to this study, increased ccf mtDNA in the serum of patients with a genetic form of PD (i.e., biallelic PD mutation PRKN/PINK1) is correlated with elevated IL-6 levels. Thus, we speculate that increased levels of the ccf mtDNA detected in the serum of PD patients could be due to massive cell death, resulting in the release of ccf- DNA as a potential signal molecule for the cytokines responsible for the described-above immunogenic response

Importantly, the level of nuclear-derived ccfDNA was also increased; however, this difference among PD and healthy controls was not as significant as it was for ccf mtDNA. 

Regarding CSF, the obtained results were in agreement with previously reported data [18,19,21] that revealed a reduced copy number of CSF ccf mtDNA in PD patients versus healthy controls. However, we found that, similarly to the serum, ccf mtDNA of PD patients significantly dominated when compared to ccfDNA, which we showed for the first time (Table 2) to reflect a possible important function of ccf mtDNA under the ccfDNA in idiopathic PD pathogenesis. This cause of the reduction of ccf mtDNA in CSF is poorly understood. PD is linked to high levels of neuronal cell death within the substantia nigra; therefore, an increased level of CSF ccf mtDNA would be expected as a consequence of the mitophagy process [8]. However, this pattern could also be caused by an overall decrease in the mitochondria pool of nerve cells, which is observed in the early stages of neurodegeneration in PD [18]. Studies that have reported decreased neuronal mtDNA copy numbers in neurodegenerative disorders have indicated that it is associated with a reduction in cell energy [40]. Reduced CSF ccf mtDNA levels in nerve cells have also been reported in other neurodegenerative diseases, such as Huntington’s disease and Alzheimer’s disease [8]. However, in Alzheimer’s disease, the observation was not confirmed by the other studies [30]. In contrast, increased CSF ccf mtDNA levels have been reported in patients with multiple sclerosis. Studies have concluded that this occurs as a direct consequence of the increased activation of inflammatory cells, which release mtDNA into the CSF [41]. 

In the case of nuclear-derived ccfDNA, we observed a decreased level in both the serum and CSF of PD patients. The mechanism driving this is unclear; however, it may be a consequence of the faster degradation of genomic DNA. Notably, ccfDNA has been reported to be more prone to nuclease degradation compared to ccf mtDNA [8]; this reflects the unknown mechanism in the CSF and serum of PD patients, which needs further study. We assumed that ccf mtDNA dominates over ccfDNA in the serum and CSF of PD patients, which may suggest some unknown mechanism in PD biogenesis in which mitochondria are engaged. 

Importantly, our study has some limitations that need to be taken into account. Firstly, there was a rather small number of participants for serum (30 PD and 15 controls), and even more so for the CSF (13 PD and five controls). It is also known that obtaining a larger sample size for serum samples of patients with PD, one of the most common neurodegenerative diseases, should not be very difficult; however, despite the relatively small serum sample size, many observed results based on the precise ddPCR method are statistically significant and novel (Table 2), and we believe that the presented data will inspire further follow-up studies.

Future study of the ccf DNA of the blood serum should also be considered. It is known that ccf-DNA that circulates in the blood originates from different tissues. Thus, this DNA has the same genome and cannot be associated with a specific source tissue through DNA sequencing [42,43,44,45,46,47]. In our study, we have shown that using target mitochondrial and nuclear genes to identify the origin of ccf-DNA in blood serum in PD patients by ddPCR enabled us to quantify ccf-DNA, which could be used in the future for optimalisation PD therapy.

It is known that Parkinson’s disease has a genetic origin (i.e., mutations in the PARK genes encoding alpha-Synuclein, DJ-1, PINK, LRRK2, etc.) in 5–10% of patients, causing so-called early-onset PD and that most of these cases are idiopathic and associated with aging. In our study, an idiopathic group of PD patients was chosen as a group reflecting various features associated with aging, and consequently, with neuroinflammation [48]. For this reason, we decided to take healthy blood donors to compare our PD results with undoubtedly homogeneous controls. It should also be noted that a previous study revealed that the ccf mtDNA of healthy individuals declined with the age of the healthy individuals [49]. In line with this finding, we performed ANOVA analysis with age as a covariate. The obtained results revealed that age may not be associated with ccf mtDNA and ccfDNA levels in PD patients versus control healthy patients, although there was no significant overall association (Figure 1e,f), (Supplementary File S2 Tables S1 and S2). 

Ccf-DNA levels vary over time depending on increased physical activity, and the existence of various medical conditions, such as infectious diseases [50]. Thus, we also determined the copy number of ccfDNA in the serum of PD patients according to gender, although this was not performed for CSF due to the smaller sample size. The obtained results revealed that the level of ccf mtDNA was significantly increased in male PD patients as compared to healthy male control patients. However, serum ccf mtDNA levels were significantly higher among the female healthy patients compared to the male healthy patients. This could suggest that the presence of hormones influences the course of this neurodegenerative disease. This observation is particularly interesting, especially when considering data obtained by Patel and Kompoliti, which showed a lower prevalence of PD among females [51]. Ultimately, we can only speculate on the influence of the hormonal or menopausal status of our female participants. Undoubtedly, the impact of the level of female hormones on PD biogenesis requires further analysis.

It is worth highlighting certain results obtained for the serum and CSF of healthy controls. For both cases, the level of ccf mtDNA was statistically higher than that of ccfDNA, and differences were more significant in the serum. This may suggest that ccf mtDNA could be engaged in unknown regulatory processes, such as cell signaling, which again illustrates the need for further study of the role of mitochondria in ccf mtDNA biogenesis.

The results of our quantitative ccf-DNA analysis of the serum and CSF of PD patients may also be correlated with some other neurodegenerative aspects, such as, for example, protein aggregation. Indeed, for ccf mtDNA, it has previously been found that there is no significant correlation between CSF ccf mtDNA and α synuclein [18].

In summary, the results obtained here recommend serum for the studies on PD patients, as it is a safer candidate for use in non-invasive diagnostic studies than CSF. Despite this and the other discussed limitations, if validated, we suggest ccf mtDNA to be used as a target for the optimization of PD therapies. 

In the future, determining the precise mechanism of ccf DNA release in PD patients would add further value to ccf mtDNA’s potential to serve as a reliable, non-invasive approach to monitoring responses to medical treatment during therapy. 

## 4. Materials and Methods

### 4.1. Collection of CSF and Serum Samples

The study was performed with the cooperation of the Department of Neurology, Division of Neurochemistry and Neuropathology and Department of Neurosurgery and Neurotraumatology, Poznan University of Medical Sciences. It was approved by the local institutional review board (206/17). Written consent forms were collected from all patients who were involved in this study.

### 4.2. Study Cohort and Sampling Procedure

The blood samples used in this study were collected from 2016 to 2020 at the University of Medical Sciences. In total, 20 healthy and 34 PD patients were included in this study. All blood and CSF samples were taken before doctoral interventions. Patients were analyzed for the assessment if they were suitable for deep brain stimulation treatment and it had been, on average, five years after their initial diagnosis of Parkinson’s disease.

Patient data, including age and length of treatment, were obtained from the patients’ pathological reports. The blood samples were processed, and the serum was isolated immediately from 1 mL of blood according to the procedure described by Al Amir Dache et al. [23]. Serum samples were immediately frozen in liquid nitrogen and stored at −80 °C. The study cohort (*n =* 30) was divided into two groups: the first group consisted of PD patients (*n =* 34), while the second consisted of healthy control patients (*n =* 20). The diagnosis of PD was established based on the criteria of the United Kingdom PD Brain Bank. The healthy controls were recruited from the Regional Blood Centre and Blood Therapy in Poznan.

Regarding the CSF samples, we collected 13 from patients diagnosed with PD and 5 from healthy controls. All samples were obtained by lumbosacral puncture and frozen and stored immediately at −20 °C.

### 4.3. ccfDNA Isolation

Ccf-DNA was isolated from both the serum and CSF samples according to the Qiagen procedure using an isolation kit specific for isolating this DNA from plasma or serum (QIAamp MinElute ccfDNA Mini Kit, Wroclaw, Poland). For each isolation procedure, 0.2 mL of serum or CSF were taken. The isolated ccf DNA was eluted in 20 μL and stored in 5 μL aliquots in standard Eppendorf tubes at −80 °C. DNA was quantified using a nanodrop spectrophotometer (Thermo Scientific, Life Technologies, Warsaw, Poland).

### 4.4. Quantification of Serum ccf mtDNA and ccfDNA Levels

DdPCR was used to quantify ccf mtDNA and ccfDNA levels. The ddPCR assay was performed using the QX200 ddPCR EvaGreen Supermix (Bio-Rad, Warsaw, Poland) [42,43]. Primers coupled with the EvaGreen dye were used to quantify fragments, with amplicons targeting the wild-type sequences of specific genes: the *KRAS* nuclear gene and the mitochondrial *COX* gene, *MT-COX3* (Table 3). Quantification of the short and long amplicons provided an estimation of the concentrations of the total ccfDNA and ccf mtDNA, respectively (Supplementary Data File S1) [23]. The mitochondrial gene encodes the mitochondrial *COX3*, while the nucleus gene is an oncogene which encodes a protein belonging to the GTPase superfamily (*KRAS*). Primer sequences for the *COX3* gene were based on previous analogue studies [23]. Primer sequences for the *KRAS* gene were designed using the PRIMER 3 program (https://bioinfo.ut.ee/primer3-0.4.0/, accessed 4 March 2019). The specificity of primers was assessed using the Primer-BLAST program (https://www.ncbi.nlm.nih.gov/tools/primer-blast/, accessed 4 March 2019), as well as in-silico PCR (https://genome.ucsc.edu/cgi-bin/hgPcr, accessed 4 March 2019). DdPCR samples were prepared following the manufacturer’s instructions. The quantitative PCR (qPCR was performed on the QuantStudio^TM^ 7Flex System (Applied Biosystems, Warsaw, Poland) [44]. For the ddPCR analysis of the serum samples, all samples were diluted 15 times. For the ddPCR analysis of the CSF samples, isolated ccf DNA was diluted five times. After preparing the correct dilutions, 1 μL of ccf DNA was added to 21 μL of the mixture, which consisted of forward and reverse primers and EvaGreen dye. The droplets were generated using the Droplet Generator (Bio-Rad). To obtain information regarding the number of copies of the *COX3* and *KRAS* genes, the QX200 Droplet Reader (Bio-Rad) was used.

Prior to the ddPCR analysis, a qPCR analysis was performed to confirm the presence of the *COX3* and *KRAS* genes and, therefore, the presence of ccf mtDNA and ccfDNA in the CSF and serum samples. The qPCR analysis allowed us to select the correct 5-fold dilution of ccf DNA for the ddPCR analysis. For qPCR analysis, all samples were diluted four times, and SYBR green dye was used.

### 4.5. Statistical Analysis

The results obtained from the ddPCR analysis were calculated using the Poisson equation. The step-by-step calculations are described below. The statistical analysis involved the Mann–Whitney U test (https://astatsa.com/WilcoxonTest/, accessed on 8 September 2021). The threshold for statistical significance was set to *p* < 0.05.

To assess the association of ccf mt DNA or ccfDNA levels with Parkinson’s disease, a multiple linear regression was conducted. Due to age heterogeneity between case and control groups, age was included in the model as a covariate to account for potential confounding effects. The regression models were formulated as (ccf mtDNA~PD + age, where ccf mtDNA or ccfDNA levels were regressed on Parkinson’s disease status (PD) and age. The analysis was performed in R (4.1.2) using the lm package. Additionally, to compare the mean levels of ccfDNA and ccf mtDNA within the control and case groups, a paired *t*-test was conducted using the *t*-test function in R. For both analyses, the significance levels were set at α = 0.05. The results were visualized using the ggplot2 package.

### 4.6. Data Presentation

The results collected from the ddPCR analysis were presented in the form of boxplot graphs generated using SigmaPlot 13.0 (Systat Software). For each generated boxplot graph, the median (solid line) and mean (dashed line) number of copies of ccf-DNA were calculated and presented. Due to the small number of PD patients and control patients tested, no outlier ccf-DNA results were removed during graph generation. Due to the small number of PD patients and control patients tested, no outlier copy number values (round, black dots above or boxplots below) were removed during graph generation.

## Figures and Tables

**Table 1:** Data from Parkinson’s disease patients in the study.

**Data of Tested Patients**			
**Serum**			
**Control Patients (*n =* 15)**		**Parkinson’s Disease Patients (*n =* 30)**	
Age			
Mean	39	Mean	62
Median	37	Median	66
Min–Max	19–54	Min–Max	40–75
Gender			
Females	4	Females	12
% of females tested	27%	% of females tested	40%
Males	11	Males	18
% of males tested	73%	% of males tested	60%
**Data of Tested Patients**			
**CSF**			
**Control Patients (*n =* 5)**		**Parkinson’s Disease Patients (*n =* 13)**	
Age			
Mean	52	Mean	57
Median	56	Median	61
Min–Max	35–69	Min–Max	37–75
Gender			
Females	3	Females	6
% of females tested	60	% of females tested	46%
Males	2	Males	7
% of males tested	40	% of males tested	54%

**Table 2:** Comparative table of previously reported results and this study regarding ccf mtDNA and ccfDNA in human blood serum and CSF. EOPD (Early-onset Parkinson’s disease); * results are not statistically significant; novel results obtained in this study are indicated in bold.

Source of Sample	Gender Male/ Female	Type of PD	Ccf mtDNA/ Healthy Control	Ccf mtDNA/ ccfDNA	Ccf DNA/ Healthy Control	Number of PD/Number of Control	Method of Analysis	Reference
serum	-	mut+/+ PD PRKN/PINK1 mut+/– PD PRKN/PINK1	increase increase	-	-	17/57 17/55	ddPCR	[21]
serum	-	idiopathic	increase	**increase**	**increase**	30/15	ddPCR	this study
serum	male	idiopathic	**increase**	increase *	no difference *	18/11	ddPCR	this study
serum	female	idiopathic	no difference *	no difference *	no difference *	12/4	ddPCR	this study
CSF	-	idiopathic	reduced	-	-	56/10	qPCR	[20]
CSF	-	EOPD	reduced	-	-	176/87	qPCR	[18]
CSF	-	idiopathic	reduced *	**increase**	increase *	13/5	ddPCR	this study

**Table 3:** Primer sequences used for ddPCR and qPCR.

Gene	Sequence of the Forward Primer (5′->3′)	Sequence of the Reverse Primer (5′->3′)
*COX3*	GACCCACCAATCACATGC	TGAGAGGGCCCCTGTTAG
*KRAS*	CCTTGGGTTTCAAGTTATATG	CCCTGACATACTCCCAAGGA

## Data Availability

Data are contained within the article and Supplementary Materials.

## Supplementary Materials

The following supporting information can be downloaded at: https://www.mdpi.com/article/10.3390/ijms25052818/s1.

## References

[1] Tolosa E., Garrido A., Scholz S.W., Poewe W. (2021). Challenges in the diagnosis of Parkinson’s disease. Lancet Neurol..

[2] Armstrong M.J., Okun M.S. (2020). Diagnosis and Treatment of Parkinson Disease. JAMA.

[3] Ranucci R. (2019). Cell-Free DNA: Applications in Different Diseases. Methods Mol. Biol..

[4] Bose A., Beal M.F. (2016). Mitochondrial dysfunction in Parkinson’s disease. J. Neurochem..

[5] Gaitsch H., Franklin R.J.M., Reich D.S. (2023). Cell-free DNA-based liquid biopsies in neurology. Brain.

[6] Rose N.C., Barrie E.S., Malinowski J., Jenkins G.P., McClain M.R., LaGrave D., Leung M.L., ACMG Professional Practice and Guidelines Committee (2022). Systematic evidence-based review: The application of noninvasive prenatal screening using cell-free DNA in general-risk pregnancies. Genet. Med..

[7] Hashimoto T., Yoshida K., Hashiramoto A., Matsui K. (2021). Cell-Free DNA in Rheumatoid Arthritis. Int. J. Mol. Sci..

[8] Gambardella S., Limanaqi F., Ferese R., Biagioni F., Campopiano R., Centonze D., Fornai F. (2019). ccf-mtDNA as a Potential Link Between the Brain and Immune System in Neuro-Immunological Disorders. Front. Immunol..

[9] Aucamp J., Bronkhorst A.J., Badenhorst C.P.S., Pretorius P.J. (2018). The diverse origins of circulating cell-free DNA in the human body: A critical re-evaluation of the literature. Biol. Rev..

[10] Thierry A.R., Messaoudi S.E., Gahan P.B., Anker P., Stroun M. (2016). Origins, structures, and functions of circulating DNA in oncology. Cancer Metastasis Rev..

[11] Wan J.C.M., Massie C., Garcia-Corbacho J., Mouliere F., Brenton J.D., Caldas C., Pacey S., Baird R., Rosenfeld N. (2017). Liquid biopsies come of age: Towards implementation of circulating tumour DNA. Nat. Rev. Cancer.

[12] Stroun M., Lyautey J., Lederrey C., Olson-Sand A., Anker P. (2001). About the possible origin and mechanism of circulating DNA apoptosis and active DNA release. Clin. Chim. Acta Int. J. Clin. Chem..

[13] Trumpff C., Michelson J., Lagranha C.J., Taleon V., Karan K.R., Sturm G., Lindqvist D., Fernström J., Moser D., Kaufman B.A. (2021). Stress and circulating cell-free mitochondrial DNA: A systematic review of human studies, physiological considerations, and technical recommendations. Mitochondrion.

[14] Tseng L.M., Yin P.H., Chi C.W., Hsu C.Y., Wu C.W., Lee L.M., Wei Y.H., Lee H.C. (2006). Mitochondrial DNA mutations and mitochondrial DNA depletion in breast cancer. Genes Chromosomes Cancer.

[15] Swarup V., Rajeswari M.R. (2007). Circulating (cell-free) nucleic acids—A promising, non-invasive tool for early detection of several human diseases. FEBS Lett..

[16] Yu M. (2012). Circulating cell-free mitochondrial DNA as a novel cancer biomarker: Opportunities and challenges. Mitochondrial DNA.

[17] Song P., Wu L.R., Yan Y.H., Zhang J.X., Chu T., Kwong L.N., Patel A.A., Zhang D.Y. (2022). Limitations and opportunities of technologies for the analysis of cell-free DNA in cancer diagnostics. Nat. Biomed. Eng..

[18] Pyle A., Brennan R., Kurzawa-Akanbi M., Yarnall A., Thouin A., Mollenhauer B., Burn D., Chinnery P.F., Hudson G. (2015). Reduced CSF mitochondrial DNA is a biomarker for early-stage Parkinson’s disease. Ann. Neurol..

[19] Lowes H., Pyle A., Santibanez-Koref M., Hudson G. (2020). Circulating cell-free mitochondrial DNA levels in Parkinson’s disease are influenced by treatment. Mol. Neurodegener..

[20] Bruno D.C.F., Donatti A., Martin M., Almeida V.S., Geraldis J.C., Oliveira F.S., Dogini D.B., Lopes-Cendes I. (2020). Circulating nucleic acids in the plasma and serum as potential biomarkers in neurological disorders. Braz. J. Med. Biol. Res..

[21] Lowes H., Kurzawa-Akanbi M., Pyle A., Hudson G. (2020). Post-mortem ventricular cerebrospinal fluid cell-free-mtDNA in neurodegenerative disease. Sci. Rep..

[22] Borsche M., König I.R., Delcambre S., Petrucci S., Balck A., Brüggemann N., Zimprich A., Wasner K., Pereira S.L., Avenali M. (2020). Mitochondrial damage-associated inflammation highlights biomarkers in PRKN/PINK1 parkinsonism. Brain.

[23] Meddeb R., Dache Z.A.A., Thezenas S., Otandault A., Tanos R., Pastor B., Sanchez C., Azzi J., Tousch G., Azan S. (2019). Quantifying circulating cell-free DNA in humans. Sci. Rep..

[24] Al Amir Dache Z., Otandault A., Tanos R., Pastor B., Meddeb R., Sanchez C., Arena G., Lasorsa L., Bennett A., Grange T. (2020). Blood contains circulating cell free respiratory competent mitochondria. FASEB J..

[25] Ludlow A.T., Robin J.D., Sayed M., Litterst C.M., Shelton D.N., Shay J.W., Wright W.E. (2014). Quantitative telomerase enzyme activity determination using droplet digital PCR with single cell resolution. Nucleic Acids Res..

[26] Huang E.E., Tedone E., O’Hara R., Cornelius C., Lai T.P., Ludlow A., Wright W.E., Shay J.W. (2017). The maintenance of telomere length in CD28+ T cells during T lymphocyte stimulation. Sci. Rep..

[27] Hindson B.J., Ness K.D., Masquelier D.A., Belgrader P., Heredia N.J., Makarewicz A.J., Bright I.J., Lucero M.Y., Hiddessen A.L., Legler T.C. (2011). Highthroughput droplet digital PCR system for absolute quantitation of DNA copy number. Anal. Chem..

[28] Robin J., Wynn J., Moscovitch M. (2016). The spatial scaffold: The effects of spatial context on memory for events. J. Exp. Psychol. Learn. Mem. Cogn..

[29] Pinheiro L.B., Coleman V.A., Hindson C.M., Herrmann J., Hindson B.J., Bhat S., Emslie K.R. (2012). Evaluation of a droplet digital polymerase chain reaction format for DNA copy number quantification. Anal. Chem..

[30] Podlesniy P., Figueiro-Silva J., Llado A., Antonell A., Sanchez-Valle R., Alcolea D., Lleo A., Molinuevo J.L., Serra N., Trullas R. (2013). Low cerebrospinal fluid concentration of mitochondrial DNA in preclinical Alzheimer disease. Ann. Neurol..

[31] Wachsmuth M., Hubner A., Li M., Madea B., Stoneking M. (2016). Age-related and heteroplasmy-related variation in human mtDNA copy number. PLoS Genet..

[32] Suárez-Rivero J.M., Pastor-Maldonado C.J., Povea-Cabello S., Álvarez-Córdoba M., Villalón-García I., Talaverón-Rey M., Suárez-Carrillo A., Munuera-Cabeza M., Sánchez-Alcázar J.A. (2021). From mitochondria to atherosclerosis. Inflamm. Path Biomed..

[33] Pyle A., Anugrha H., Kurzawa-Akanbi M., Yarnall A., Burn D., Hudson G. (2016). Reduced mitochondrial DNA copy number is a biomarker of Parkinson’s disease. Neurobiol. Aging.

[34] West A.P., Shadel G.S. (2017). Mitochondrial DNA in innate immune responses and inflammatory pathology. Nat. Rev. Immunol..

[35] Regner A., Meirelles L.D.S., Ikuta N., Cecchini A., Simon D. (2018). Prognostic utility of circulating nucleic acids in acute brain injuries. Expert Rev. Mol. Diagn..

[36] Riley J.S., Tait S.W. (2020). Mitochondrial DNA in inflammation and immunity. EMBO Rep..

[37] Kigerl K.A., de Rivero Vaccari J.P., Dietrich W.D., Popovich P.G., Keane R.W. (2014). Pattern recognition receptors and central nervous system repair. Exp. Neurol..

[38] Decout A., Katz J.D., Venkatraman S., Ablasser A. (2021). The cGAS-STING pathway as a therapeutic target in inflammatory diseases. Nat. Rev. Immunol..

[39] Dib B., Lin H., Maidana D.E., Tian B., Miller J.B., Bouzika P., Miller J.W., Vavvas D.G. (2015). Mitochondrial DNA has a pro-inflammatory role in AMD. Biochim. Biophys. Acta.

[40] Keeney P.M., Bennett J.P. (2010). Interestingly ALS spinal neurons show varied and reduced mtDNA gene copy numbers and increased mtDNA gene deletions. Mol. Neurodegener..

[41] Frank M.O. (2016). Circulating cell-free DNA differentiates severity of inflammation. Biol. Res. Nurs..

[42] Lehmann-Werman R., Magenheim J., Moss J., Neiman D., Abraham O., Piyanzin S., Zemmour H., Fox I., Dor T., Grompe M. (2018). Monitoring liver damage using hepatocyte-specific methylation markers in cell-free circulating DNA. JCI Insight.

[43] Lehmann-Werman R., Neiman D., Zemmour H., Moss J., Magenheim J., Vaknin-Dembinsky A., Rubertsson S., Nellgård B., Blennow K., Zetterberg H. (2016). Identification of tissue-specific cell death using methylation patterns of circulating DNA. Proc. Natl. Acad. Sci. USA.

[44] Zemmour H., Planer D., Magenheim J., Moss J., Neiman D., Gilon D., Korach A., Glaser B., Shemer R., Landesberg G. (2018). Non-invasive detection of human cardiomyocyte death using methylation patterns of circulating DNA. Nat. Commun..

[45] Sun K., Jiang P., Chan K.C., Wong J., Cheng Y.K., Liang R.H., Chan W.K., Ma E.S., Chan S.L., Cheng S.H. (2015). Plasma DNA tissue mapping by genome-wide methylation sequencing for noninvasive prenatal, cancer, and transplantation assessments. Proc. Natl. Acad. Sci. USA.

[46] Moss J., Magenheim J., Neiman D., Zemmour H., Loyfer N., Korach A., Samet Y., Maoz M., Druid H., Arner P. (2018). Comprehensive human cell-type methylation atlas reveals origins of circulating cell-free DNA in health and disease. Nat. Commun..

[47] Dor Y., Cedar H. (2018). Principles of DNA methylation and their implications for biology and medicine. Lancet.

[48] Pajares M., IRojo A., Manda G., Boscá L., Cuadrado A. (2020). Inflammation in Parkinson’s Disease: Mechanisms and Therapeutic Implications. Cells.

[49] Lazo S., Noren Hooten N., Green J., Eitan E., Mode N.A., Liu Q.R., Zonderman A.B., Ezike N., Mattson M.P., Ghosh P. (2021). Mitochondrial DNA in extracellular vesicles declines with age. Aging Cell.

[50] Sherwood K., Weimer E.T. (2018). Characteristics, properties, and potential applications of circulating cell-free dna in clinical diagnostics: A focus on transplantation. J. Immunol. Methods.

[51] Patel R., Kompoliti K. (2023). Sex and Gender Differences in Parkinson’s Disease. Neurol. Clin..

