# Supplementary material for: Quantification of Circulating Cell-Free DNA in Idiopathic Parkinson’s Disease Patients
Source: Int J Mol Sci. 2024 Feb 29;25(5):2818. doi: 10.3390/ijms25052818 (PMC10931594; doi:10.3390/ijms25052818)
Supplement: Supplementary file 1 [file ijms-25-02818-s001.zip › Supplementary File S2.pdf]

Supplementary Table S1. Results of regression of Parkinson's disease presence (PD) and age on ccf mtDNA levels.

Residuals				
Min	1Q	Median	3Q	Max
-382.1	-155	-21.1	102.4	636.5
Coefficients				
	Estimate	SE	t	p-value
Intercept	415.396	249.335	1.666	0.1032
PD	231.872	121.552	1.908	0.0633
Age	1.424	3.931	0.362	0.719
Residual standard error: 253.2 on 42 degrees of freedom				
Multiple R-squared 0.2088, Adjusted R-square 0.1711				
F-statistic 5.542 on 2 and 42 DF, p-value 0.007309				

Supplementary Table S2. Results of regression of Parkinson's disease presence (PD) and age on nu mtDNA levels.

Residuals				
Min	1Q	Median	3Q	Max
-27.78	-9.297	-3.130	6.833	60.666
Coefficients				
	Estimate	SE	t	p-value
Intercept	19.7135	16.4209	1.201	0.237
PD	7.7661	8.0053	-0.970	0.338

Age	0.1257	0.2589	0.486	0.630
Residual standard error : 16.68 on 42 degrees of freedom				
Multiple R-squared 0.09379, Adjusted R-square 0.05063				
F-statistic 2.173 on 2 and 42 DF, p-value 0.1264				
